# Electrostatic Mis-Interactions Cause Overexpression Toxicity of Proteins in *E. coli*

**DOI:** 10.1371/journal.pone.0064893

**Published:** 2013-05-29

**Authors:** Gajinder Pal Singh, Debasis Dash

**Affiliations:** G. N. Ramachandran Knowledge Center for Genome Informatics, Institute of Genomics and Integrative Biology (Council of Scientific and Industrial Research), Delhi, India; CRG, Spain

## Abstract

A majority of *E. coli* proteins when overexpressed inhibit its growth, but the reasons behind overexpression toxicity of proteins remain unknown. Understanding the mechanism of overexpression toxicity is important from evolutionary, biotechnological and possibly clinical perspectives. Here we study sequence and functional features of cytosolic proteins of *E. coli* associated with overexpression toxicity to understand its mechanism. We find that number of positively charged residues is significantly higher in proteins showing overexpression toxicity. Very long proteins also show high overexpression toxicity. Among the functional classes, transcription factors and regulatory proteins are enriched in toxic proteins, while catalytic proteins are depleted. Overexpression toxicity could be predicted with reasonable accuracy using these few properties. The importance of charged residues in overexpression toxicity indicates that nonspecific electrostatic interactions resulting from protein overexpression cause toxicity of these proteins and suggests ways to improve the expression level of native and foreign proteins in *E. coli* for basic research and biotechnology. These results might also be applicable to other bacterial species.

## Introduction

Expression levels of proteins can be highly optimized in bacterial cells to maximize fitness [1], but it may be desirable in lab to increase the expression level of proteins beyond their normal cellular levels, which often leads to growth inhibition [2]. Protein overexpression in model organism *Escherichia coli* is utilized in biophysical, biochemical, structural studies of proteins, production of industrial important enzymes [3] and development of strains for producing metabolites [4], biofuel [5] and bioremediation [6]. Furthermore, gene duplication and hence protein overexpression is also important from evolutionary and clinical perspective, where it can lead to novel phenotypes including antibiotic resistance [7], [8]. Hence it is important to understand the mechanism of overexpression toxicity of proteins in *E. coli*.

In yeast, overexpression library of endogenous proteins has been described [9]. Overexpression leads to reduction in the growth rate in a subset (∼15%) of proteins, which were highly enriched in structural disorder [10]. Disordered regions and proteins in eukaryotes are widely associated with protein-protein and protein-DNA interactions [11]–[13], so their increased levels may lead to large number of promiscuous interactions [10] and thus toxicity. Disorder was also found to be associated with overexpression toxicity in other eukaryotes: *Drosophila melanogaster* and *C. elegans*, and with dosage sensitive oncogenes in mice and human [10]. In addition to disordered proteins, highly expressed proteins and members of protein complexes are highly sensitive to fold increase from their normal levels [14]. *E. coli*, like most bacteria have few disordered regions and proteins, thus disordered regions mediated promiscuous interactions could not be the major mechanism of overexpression toxicity in bacteria.

An overexpression library has been described in *E. coli* called ASKA library (**A** Complete **S**et of *E. coli* **K**-12 ORF **A**rchive) in which most of its ORFs have been individually cloned with histidines and seven spacer amino acids at the N-terminal end, and five spacer amino acids and GFP (Green Fluorescent Protein) at the C-terminal end in IPTG inducible, expression vector [2]. Effect on growth and GFP fluorescence by IPTG induction was examined for each of the clone and classified into three categories each (“almost no growth”, “slow growth”, “normal growth” and “high fluorescence”, “fluorescence”, “no fluorescence” respectively). Under these conditions, majority of proteins inhibit the growth of *E. coli* when overexpressed, while overexpression of a subset of proteins leads to severe toxicity. Particularly, membrane proteins are highly toxic on overexpression [2]. Here we study sequence and functional properties of cytoplasmic proteins of *E. coli* which are highly toxic on overexpression to understand its mechanism and find that number of positively charged residues to be the most important feature of toxic proteins. Functional classes also show differential enrichment: transcription factors and regulatory proteins were overrepresented, while catalytic proteins were underrepresented in toxic proteins.

## Results

**Figure 1:**
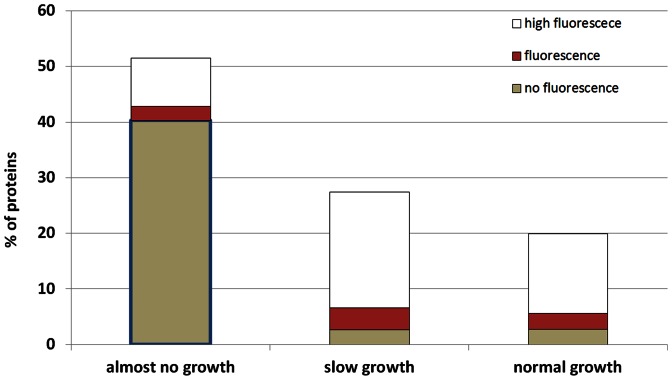
Effects on growth and GFP fluorescence of proteins on overexpression. Protein overexpression was induced by adding IPTG to ASKA clones grown in LB medium at 37C, and effects on growth and GFP fluorescence was classified into three categories each (“almost no growth”, “slow growth”, “normal growth” and “high fluorescence”, “fluorescence”, “no fluorescence” respectively) [2]. We defined “toxic” proteins as those classified as “almost no growth” and “no fluorescence” (marked with blue outline). Overall 40% of clones fall into this category (1589/3956).

Protein overexpression upon IPTG induction (37C, LB) of the ASKA library leads to growth inhibition in about 79% of clones (52% “almost no growth” +27% “slow growth”), Figure 1. In “almost no growth” class a small fraction of clones do show GFP fluorescence (Figure 1), indicating some growth. Since we were interested in proteins whose overexpression is most toxic to *E. coli*, thus even small overexpression is likely to cause growth inhibition, we defined “toxic” proteins as those classified as “almost no growth” and “no fluorescence”. Overall 40% (1589/3956) of the clones fall into this category. Rest 60% proteins were labeled as “non-toxic”.

### High toxicity of membrane and periplasmic proteins

**Figure 2:**
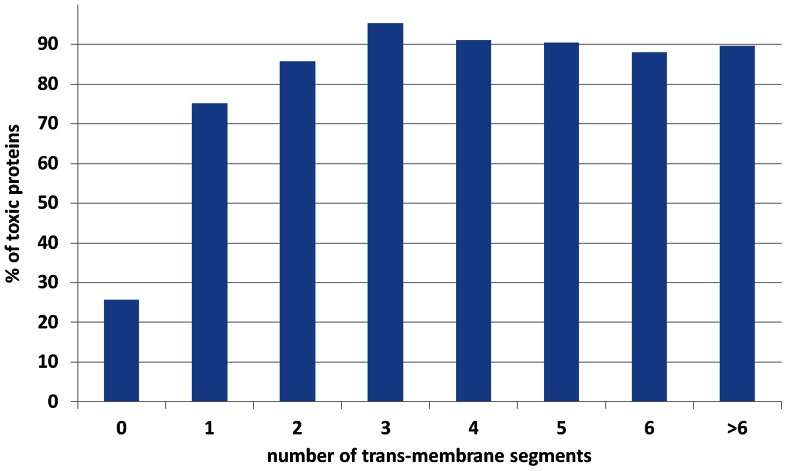
Percentage of toxic proteins as a function of number of trans-membrane segments. In proteins without any trans-membrane segment, about 25% are toxic. This percentage increases to ∼73% in proteins with one trans-membrane segment and ∼85% in proteins with two or more trans-membrane segments. Number of trans-membrane segments were predicted using TMHMM [35].

Membrane proteins are known to be highly toxic when overexpressed [2]. About 85% of proteins with at least one predicted trans-membrane segment are toxic. This fraction increases further to 89% in proteins with two or more trans-membrane segments (Figure 2). With respect to localization, outer membrane proteins and periplasmic proteins are also very toxic (83% and 72% respectively), even though they rarely have predicted trans-membrane regions indicating that extreme toxicity is a general property of secretory proteins, not just proteins with trans-membrane segments. These results are consistent with the hypothesis that saturation of Sec translocation machinery (the major membrane translocation machinery in *E. coli*) by overexpression of secretory proteins is responsible for their extreme toxicity [15].

Considering the high and potentially different mechanism of toxicity of secretory from cytoplasmic proteins, we excluded membrane (outer and inner membrane) and periplasmic proteins from all further analyses, which leave 2444 proteins, 432 of which are toxic.

### Sequence features associated with toxicity

**Figure 3:**
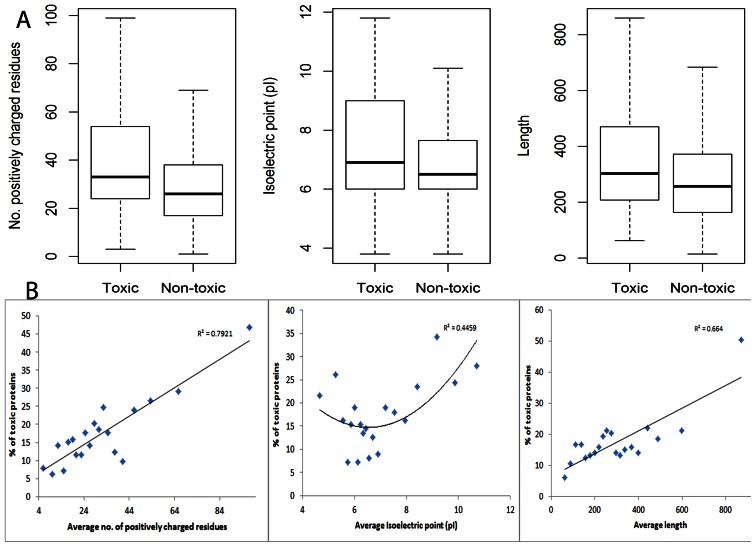
Sequence features associated with toxicity. (A) Toxic proteins have on average higher number of positively charged residues (arginine and lysine), isoelectric point (pI) and length than non-toxic proteins. Wilcox-test *p* values are 2e-17, 6e-4 and 5e-10 respectively. (B) Proteins are binned into equal sized 20 bins (thus each bin has 5% of proteins) and percentage of proteins which are toxic is plotted for each bin as a function of three sequence features. Linear regression lines are plotted for average positively charged residues and average length and quadratic regression is plotted for pI.

To better understand the mechanism of toxicity of cytoplasmic proteins, we considered number of sequence features for their relationship with toxicity. On average, toxic proteins were found to have significantly higher number of positively (arginine and lysine) charged amino acid residues, are longer and have extreme isoelectric point (pI) (Figure 3a and Figure 3b). Number of positively charged residues is the most important feature associated with toxicity of proteins (Figure 3). The effect of length is only evident for very long proteins (Figure 3b). Significantly higher number of positively charged residues in toxic proteins indicates that electrostatic mis-interactions resulting from protein overexpression is an important cause of toxicity in *E. coli*.

### Functional classes associated with toxicity

**Figure 4:**
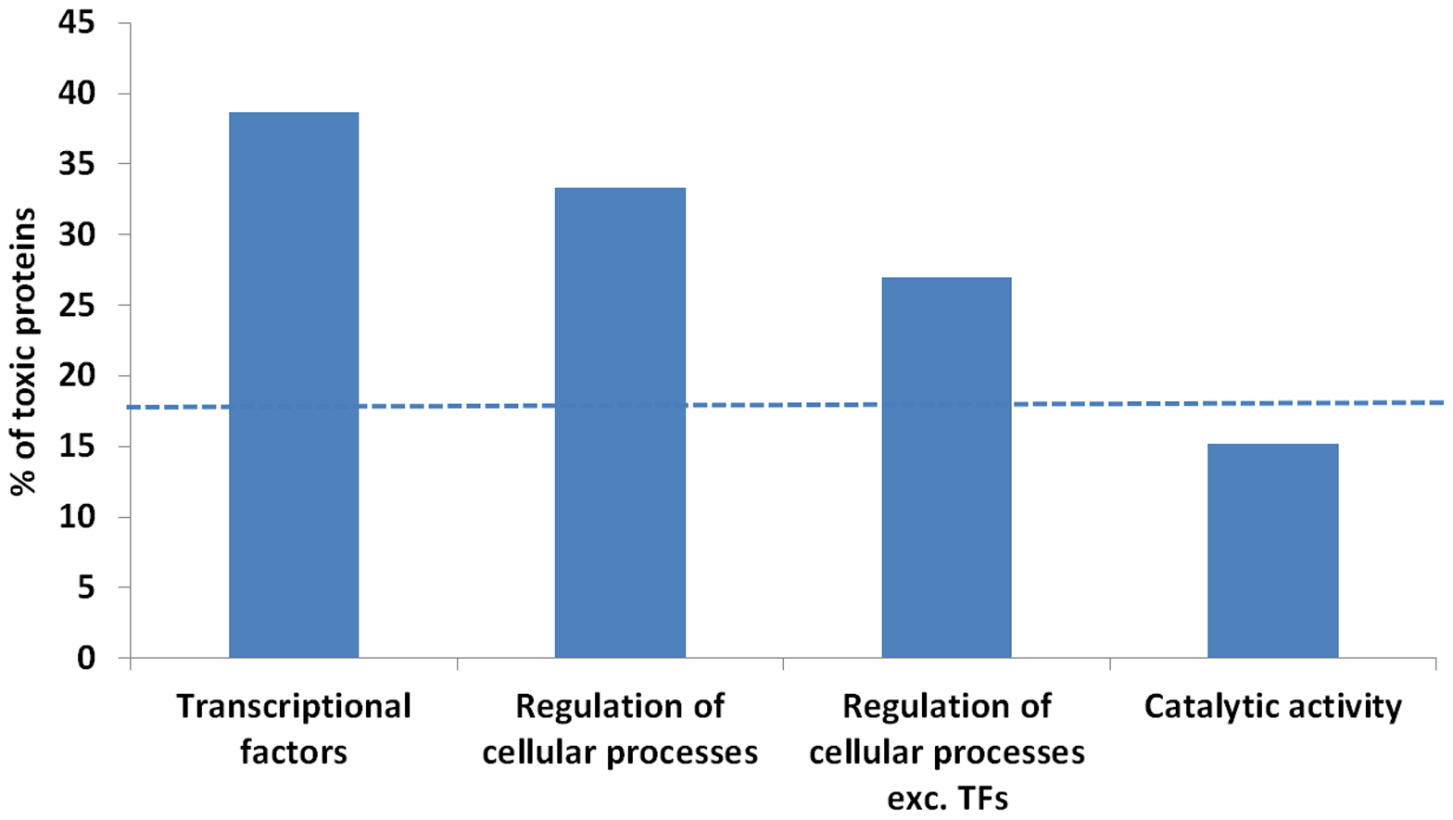
Functional classes associated with toxicity. Percentage of toxic proteins is much higher in transcription factors (Fisher *p* = 1e-13) and in “regulation of cellular processes” (Fisher *p* = 1e-13). “regulation of cellular processes” was enriched in toxic proteins even after excluding transcription factors (Fisher *p* = 3e-5). Catalytic proteins were significantly depleted in toxic proteins (Fisher *p* = 3e-4). Dotted line indicates overall average in cytoplasmic proteins.

Next we analyzed functional classes significantly associated with toxic proteins. We considered higher level GO classes in which about 200 or more proteins were present (18 functional classes). Functional classes significantly overrepresented in toxic proteins are “nucleic acid binding transcription factor activity” and “regulation of cellular processes”, while the class significantly underrepresented is “catalytic activity” (Figure 4). Since many regulatory proteins are also transcription factors, we analyzed whether regulatory proteins excluding transcription factors are also enriched in toxic proteins. Excluding transcription factors, “regulation of cellular processes”, is still enriched in toxic proteins (Figure 4), suggesting that toxicity is associated with dysregulation of cellular processes in general.

### Predictive accuracy and independence of sequence and functional features

**Figure 5:**
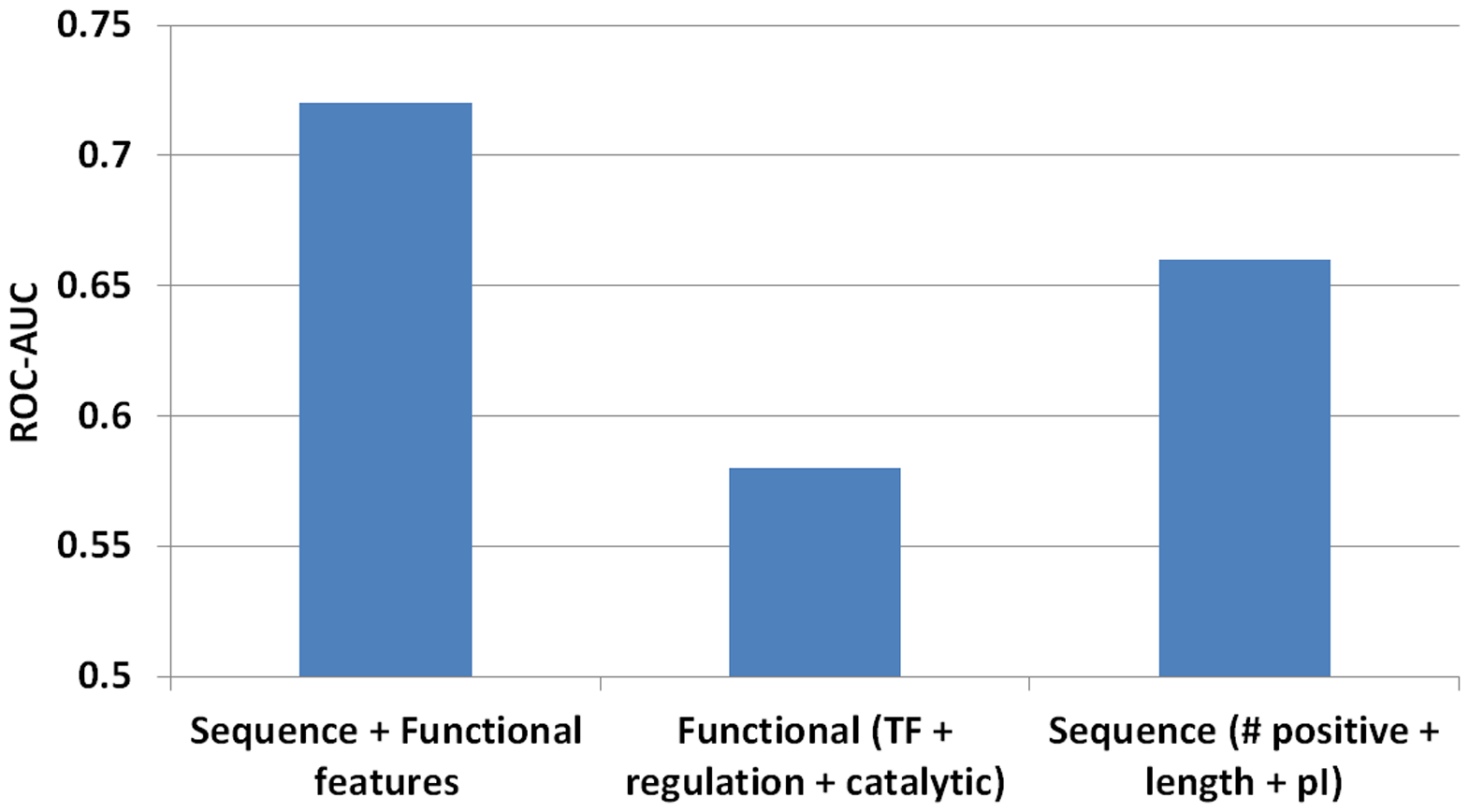
Independence of sequence and functional properties in predicting toxicity. Prediction accuracy (ROC-AUC) of overexpression toxicity from sequence (positively charged residue count, pI, and length) and functional features (transcription factor, regulation and catalytic function information). Combining sequence and functional features increases the predictive power indicating at least their partial independence. TF = transcription factors, pI = Isoelectric point.

In order to assess the predictive power and independence of sequence and functional features identified, we build a Random Forest model [16]. Using positively charged residue count, pI, length, transcription factor, regulatory and catalytic function information, the model can predict toxicity with area under receiver operating characteristic curve (ROC-AUC) of 0.72 (Figure S1), showing that these few features have enough information to predict protein toxicity with reasonable accuracy. A random predictor would have ROC-AUC of 0.5, while a perfect predictor would have ROC-AUC of 1. Functional classes (transcription factor, regulatory and catalytic function information) alone predict toxicity with ROC-AUC of 0.58, while sequence features (positively charged residue count, pI, and length) alone predict toxicity with ROC-AUC of 0.67. Increase in accuracy by adding functional and sequence features (Figure 5) indicate at least partial independence of these features in predicting toxicity.

## Discussion

Here we analyze a number of sequence and functional properties associated with proteins that show overexpression toxicity in *E. coli* to understand its mechanism. While membrane proteins are known to be highly toxic when overexpressed [2], we find that periplasmic proteins, which generally do not have trans-membrane segments, also show very high toxicity. The Sec pathway is the major route of protein translocation across and insertion into inner membrane of *E. coli*. The fact that most secretory proteins show very high toxicity is consistent with the hypothesis that saturation of Sec translocation machinery by overexpression of secretory proteins is responsible for their extreme toxicity [15]. Considering the high and potentially different mode of toxicity of secretory proteins, we focused on the mechanism of toxicity of cytoplasmic proteins.

While a number of studies have analyzed sequence features associated with overexpression of soluble proteins in *E. coli* and bacterial cell-free systems [17]–[27], none has examined the sequence and functional features associated with overexpression of endogenous proteins on the growth of *E. coli*. We find that number of positively charged residues is the most predictive feature of overexpression toxicity (Figure 3) of cytoplasmic proteins. Toxic proteins have significantly higher isoelectric point overall (Figure 3a), though proteins with very low isoelectric point are also more toxic (Figure 3b). These results indicate that electrostatic mis-interactions induced by increased concentration mediate toxicity of cytoplasmic proteins in *E. coli*. Toxic proteins were also significantly longer; particularly very large proteins (top 5% in length, Figure 3b) were highly toxic. The larger surface area of longer proteins may allow more mis-interactions.

Misfolding and self-aggregation (inclusion bodies) is commonly observed during protein overexpression in *E. coli* and may be toxic [28]. However higher charge on proteins is often associated with increased solubility and lower self-aggregation [17], [19]–[21], [23], [25]–[27], [29], suggesting that misfolding and self-aggregation is not the major mechanism of overexpression toxicity. Indeed *in vitro* protein solubility information [25] did not increase prediction accuracy of the random forest model trained on length, pI and number of positively charged residues. Furthermore, toxic proteins do not have higher hydrophobicity than non-toxic proteins (mean hydrophobicity 0.472 vs. 0.475 respectively, two tailed t-test *p* = 0.03), which is often associated with self-aggregation. Chaperone (GroEL) substrates [30], [31] are also not enriched in toxic proteins (Fisher *p* = 0.5).

It is tempting to speculate that high toxicity of positively charged proteins is due to their interactions with negatively charged DNA, which may cause transcription dysregulation (also see below) preventing the expression of essential proteins. The larger surface area of longer proteins may allow more mis-interactions. The importance of charged residues in protein sequence for toxicity suggests that reducing the charged residues (particularly positively charged residues) may reduce the overexpression toxicity. This could be done by removing charged stretches from the protein or making site directed mutagenesis. Reducing the length of the protein in cases where protein is very long (e.g. cloning different domains separately) may also be useful in decreasing overexpression toxicity. While we have used simple measures of charge of the protein, utilizing more sophisticated features that take into account the distribution of charged residues on the sequence and structure of the protein may allow better prediction of toxicity and may also be useful in designing of antimicrobial peptides, whose activity is attributed to their charge [32].

In the functional classes, transcription factors are highly toxic on overexpression (Figure 4). Transcription factors have only marginally higher positively charged residues than non-transcription factors (median 30 vs. 27 respectively, Wilcox *p* 0.03) and are not different in length (median 264 vs. 265 respectively, Wilcox *p* 0.8), thus this effect is not dependent on these features. We hypothesize that overexpression of transcription factors may allow them to bind to non-native DNA sites, which may saturate the transcription machinery and prevent transcription of proteins important for cell survival. Regulatory proteins excluding transcription factors were also enriched in toxic proteins, though less than transcription factors (Figure 4). Overexpression of regulatory proteins may also eventually cause transcription dysregulation leading to growth inhibition. Catalytic proteins is an interesting class because it shows significantly less toxicity despite the fact that these have significantly higher positively charged residues than non-catalytic proteins (median 33 vs. 21 respectively, Wilcox *p* 3e-74) and are longer (median 327 vs. 180 respectively, Wilcox *p* 5e-113). As expected, within catalytic proteins, toxic proteins have significantly higher positively charged residues than non-toxic proteins (median 48 vs. 31 respectively, Wilcox *p* 3e-20) and are longer (median 430 vs. 315 respectively, Wilcox *p* 1e-17). At present it is unclear as to why catalytic proteins are less sensitive to overexpression toxicity than non-catalytic proteins.

Dosage balance hypothesis posits that imbalance in the relative amount of proteins in protein complex (over/under expression) would disrupt its functionality [33]. Thus complexes should be enriched in toxic proteins. While we find that “macromolecular complexes” are enriched in toxic proteins (28% toxic proteins in “macromolecular complexes” vs. 17% in rest, Fisher *p* = 1e-4), these proteins also have significantly more positively charged residues (Wilcox *p* = 8e-5). Further, adding protein complex information did not increase the predictive power of random forest model. These observations suggest that enhanced toxicity of proteins in complexes is also due to electrostatic mis-interactions rather than dosage imbalance.

How does mechanism of overexpression toxicity compare between yeast and *E. coli*? In yeast, proteins showing overexpression toxicity are highly enriched in structural disorder, which is widely associated with protein-protein and protein-DNA interactions in eukaryotes [11]–[13], so their increased levels may lead to large number of promiscuous interactions [10] and toxicity. *E. coli*, like most bacteria have few disordered regions and proteins, so *a priori* it might be expected that mechanism of overexpression toxicity be very different in *E. coli* and yeast. However, we find that in *E. coli*, sequence features associated with promiscuous electrostatic interactions are significantly associated with overexpression toxicity. These results show that basic mechanism of overexpression toxicity by mis-interactions is common between yeast and *E. coli* (and hence elephants [34]), suggesting that this may be a universal phenomenon.

## Materials and Methods

The development of ASKA library is described by Kitagawa *et al.* [2]. Data on overexpression toxicity of proteins was downloaded from http://ecoli.naist.jp/GB8-dev/index.jsp?page=resource_download.jsp. Trans-membrane segments were predicted using TMHMM [35]. Gene ontology class and localization information (“membrane”, “outer membrane” and “periplasmic space”) was obtained from ECOCYC database [36]. For functional analyses we considered all GO function and process classes with about 200 or more proteins. There were 18 such classes. Protein hydrophobicity was calculated with Kyte and Doolittle hydrophobicity scale normalized from 0 to 1.

We used Random forest to test the predictive power and independence of sequence and functional features. Random forest is a statistical learning algorithm that uses an ensemble of decision trees [16], [37]. In random forests, prediction error is estimated internally without the need for explicit cross-validation as each decision tree is constructed using a different bootstrap sample of the original data and approximately one-third of the cases are left out from the training sample and not used in the construction of the tree. Thus, these left-out cases can be used to estimate prediction error. As number of toxic proteins was much smaller than non-toxic proteins, we randomly selected equal number of non-toxic proteins to build the classifier. This was done 10 times and average area under receiver operating characteristic curve (ROC-AUC) is reported as an accuracy measure.

## Supporting Information

Figure S1 **ROC curve illustrating the accuracy of toxicity prediction based on sequence and functional features.** Considering all sequence (positively charged residue count, pI, and length) and functional features (transcription factor, regulation and catalytic function information), the area under the ROC curve is 0.72.
(TIFF)
