# Supplementary figures and images for: Electrostatic Mis-Interactions Cause Overexpression Toxicity of Proteins in E. coli
Source: PLoS One. 2013 May 29;8(5):e64893. doi: 10.1371/journal.pone.0064893 (PMC3667126; doi:10.1371/journal.pone.0064893)

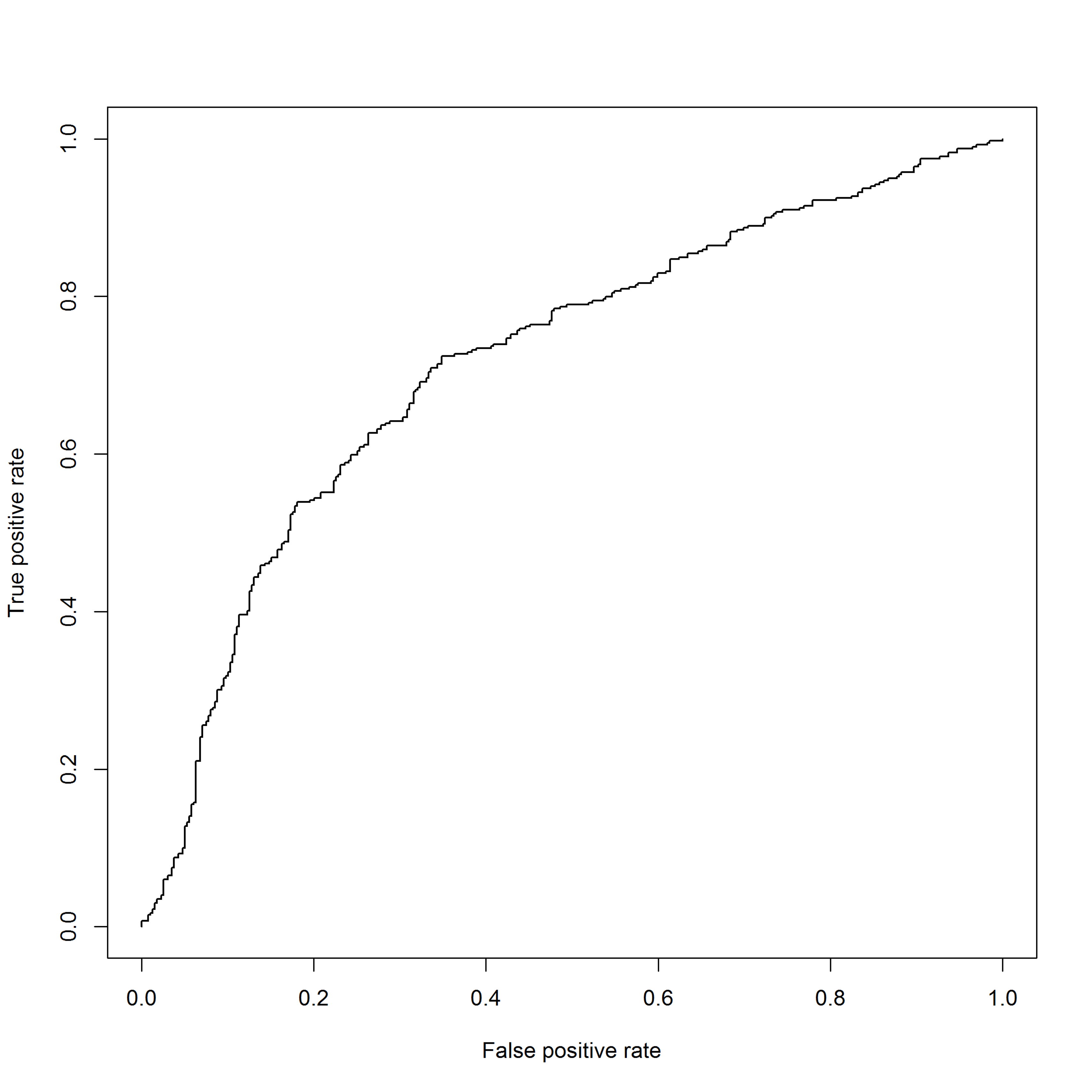

Supplement: Figure S1 — ROC curve illustrating the accuracy of toxicity prediction based on sequence and functional features. Considering all sequence (positively charged residue count, pI, and length) and functional features (transcription factor, regulation and catalytic function information), the area under the ROC curve is 0.72. (TIFF) [file pone.0064893.s001.tiff]
